# Molecular Epidemiology of SARS-CoV-2 in Greece Reveals Low Rates of Onward Virus Transmission after Lifting of Travel Restrictions Based on Risk Assessment during Summer 2020

**DOI:** 10.1128/mSphere.00180-21

**Published:** 2021-06-30

**Authors:** Evangelia Georgia Kostaki, Georgios A. Pavlopoulos, Kleio-Maria Verrou, Giannis Ampatziadis-Michailidis, Vaggelis Harokopos, Pantelis Hatzis, Panagiotis Moulos, Nikolaos Siafakas, Spyridon Pournaras, Christos Hadjichristodoulou, Fani Chatzopoulou, Dimitrios Chatzidimitriou, Periklis Panagopoulos, Panagiota Lourida, Aikaterini Argyraki, Theodoros Lytras, Spyros Sapounas, Gerasimos Gerolymatos, Georgios Panagiotakopoulos, Panagiotis Prezerakos, Sotirios Tsiodras, Vana Sypsa, Angelos Hatzakis, Cleo Anastassopoulou, Nikolaos Spanakis, Athanasios Tsakris, Meletios Athanasios Dimopoulos, Anastasia Kotanidou, Petros Sfikakis, Georgios Kollias, Gkikas Magiorkinis, Dimitrios Paraskevis

**Affiliations:** a Department of Hygiene, Epidemiology and Medical Statistics, Medical School, National and Kapodistrian University of Athens, Athens, Greece; b Center of New Biotechnologies & Precision Medicine, Medical School, National and Kapodistrian University of Athens, Athens, Greece; c Institute for Fundamental Biomedical Research, Biomedical Sciences Research Center “Alexander Fleming,” Vari, Greece; d Laboratory of Clinical Microbiology, ATTIKON University Hospital, Medical School, National and Kapodistrian University of Athens, Athens, Greece; e Laboratory of Hygiene and Epidemiology, Faculty of Medicine, Larisa, Greece; f Labnet, Laboratories, Thessaloniki, Greece; g Department of Microbiology, Medical School, Aristotle University of Thessaloniki, Thessaloniki, Greece; h 2nd Department of Internal Medicine, General Hospital of Alexandroupoli, Democritus University of Thrace, Alexandroupoli, Greece; i Infectious Diseases Clinic A, Thoracic Diseases General Hospital Sotiria, Athens, Greece; j School of Medicine, European University Cyprus, Nicosia, Cyprus; k National Public Health Organization, Athens, Greece; l Department of Nursing, University of Peloponnese, Tripoli, Greece; m 4th Department of Internal Medicine, Attikon University Hospital, Medical School, National and Kapodistrian University of Athens, Athens, Greece; n Department of Microbiology, Medical School, National and Kapodistrian University of Athens, Athens, Greece; o Department of Therapeutics, Medical School, National and Kapodistrian University of Athens, Athens, Greece; p 1st Intensive Care Unit, General Hospital Evangelismos, National and Kapodistrian University of Athens, Athens, Greece; q 1st Department of Propaedeutic Internal Medicine, Medical School, National and Kapodistrian University of Athens, Athens, Greece; r Institute for Bioinnovation, Biomedical Sciences Research Center “Alexander Fleming,” Vari, Greece; s Department of Physiology, Medical School, National and Kapodistrian University of Athens, Athens, Greece; U.S. Centers for Disease Control and Prevention

**Keywords:** SARS-CoV-2, molecular epidemiology, public health, phylogeography, travel restrictions

## Abstract

The novel coronavirus severe acute respiratory syndrome coronavirus 2 (SARS-CoV-2) spread rapidly during the first months of 2020 and continues to expand in multiple areas across the globe. Molecular epidemiology has provided an added value to traditional public health tools by identifying SARS-CoV-2 clusters or providing evidence that clusters based on virus sequences and contact tracing are highly concordant. Our aim was to infer the levels of virus importation and to estimate the impact of public health measures related to travel restrictions to local transmission in Greece. Our phylogenetic and phylogeographic analyses included 389 full-genome SARS-CoV-2 sequences collected during the first 7 months of the pandemic in Greece and a random collection in five replicates of 3,000 sequences sampled globally, as well as the best hits to our data set identified by BLAST. Phylogenetic trees were reconstructed by the maximum likelihood method, and the putative source of SARS-CoV-2 infections was inferred by phylogeographic analysis. Phylogenetic analyses revealed the presence of 89 genetically distinct viruses identified as independent introductions into Greece. The proportion of imported strains was 41%, 11.5%, and 8.8% during the three periods of sampling, namely, March (no travel restrictions), April to June (strict travel restrictions), and July to September (lifting of travel restrictions based on thorough risk assessment), respectively. The results of phylogeographic analysis were confirmed by a Bayesian approach. Our findings reveal low levels of onward transmission from imported cases during summer and underscore the importance of targeted public health measures that can increase the safety of international travel during a pandemic.

**IMPORTANCE** Our study based on current state-of-the-art molecular epidemiology methods suggests that virus screening and public health measures after the lifting of travel restrictions prevented SARS-CoV-2 onward transmission from imported cases during summer 2020 in Greece. These findings provide important data on the efficacy of targeted public health measures and have important implications regarding the safety of international travel during a pandemic. Our results can provide a roadmap about prevention policy in the future regarding the reopening of borders in the presence of differences in vaccination coverage, the circulation of the virus, and the presence of newly emergent variants across the globe.

## INTRODUCTION

In December 2019, a new respiratory disease was reported in Wuhan, China, which was found to be caused by a novel coronavirus named severe acute respiratory syndrome coronavirus 2 (SARS-CoV-2) (1). The new virus spread globally and caused a pandemic associated with increased morbidity and mortality rates (2). In the absence of an effective vaccine in the first year of the pandemic, nonpharmaceutical interventions (NPIs), such as social distancing, use of masks in the community, travel restrictions, and school and nonessential shop closures were implemented to control community transmission (3). The health, social, and economic consequences of the pandemic are continuing; thus, a better understanding of the characteristics of SARS-CoV-2 transmission is needed to minimize its consequences.

Molecular epidemiology analyses of SARS-CoV-2 full-genome sequences have been extensively performed to classify viral diversity into groups or lineages/sublineages (4), to provide continuous monitoring of virus dispersal patterns, and to obtain insights into critical epidemiological or public health issues related to the geographic origin and dating of viral transmission (5–7). For example, the results of phylogenetic studies revealed that the origin of transmission during the first pandemic wave in Italy and in Seattle, Washington, were from different sources in Asia (8). Phylogenetic analysis of virus samples revealed SARS-CoV-2 clusters and tourism-associated virus dispersal of the first wave in Austria (9). In the United Kingdom, where virus genetic diversity has been systematically surveyed (5), a detailed description of the characteristics of transmission by means of the number and size of local clusters has been performed, as well as quantification of the spatiotemporal characteristics of viral diversity (10). The origin and dynamics of virus importation patterns during the first wave in the United Kingdom were also mapped (10). Importantly, a study from Iceland reported high concordance between the contacts identified by contact tracing and molecular data, suggesting that the latter can be used to control viral transmission in the community (11). Recently, genomic surveillance has been of interest due to reports that certain new lineages found to rapidly spread across the United Kingdom, South Africa, Brazil, and other areas in recent months (i.e., B.1.1.7, B.1.351, and P.1) may confer different biological characteristics to the virus (12–14).

In Greece, the first pandemic wave was mild due to the early implementation of public health measures and the high compliance of the population with the imposed lockdowns (15, 16). Public health measures included the suspension of all educational activities and all services of religious worship, and the closure of bars, cafes, restaurants, retail shops, museums, and sports facilities in the country (https://eody.gov.gr). Moreover, a travel ban for travellers coming from abroad and a 14-day posttravel quarantine period were implemented in the middle of March. It has been shown that, during this period, an outbreak in Athens, Greece, was seeded from different countries (17). By the end of October 2020, the country was experiencing rapid increases in the number of SARS-CoV-2 cases in the metropolitan area of Thessaloniki and other areas of northern Greece. In the meantime, between the lifting of the first measures in May and this second wave, the number of cases remained relatively low, even after travel restrictions were lifted at the beginning of July 2020. To date, several issues remain unanswered, such as how SARS-CoV-2 was introduced in the country at different time periods, what the patterns of virus dispersal were, and importantly, what the impact of the lifting of travel restrictions was on SARS-CoV-2 transmission.

By applying molecular epidemiology methods, we aimed to quantify the levels of virus importation in comparison with surveillance data during these different time periods, to investigate the patterns of SARS-CoV-2 dispersal, and to estimate the impact of public health measures related to travel restrictions to local transmission in Greece.

## RESULTS

Our study data comprised of 389 unique full-genome SARS-CoV-2 sequences, of which 280 were newly generated and 109 were available on the GISAID database, collected in Attica, Greece, until 1 December 2020 (17). The vast majority of our samples had been collected in Attica (*n* = 353, 90.7%), which is the largest metropolitan area and comprises approximately 40% of the population in Greece and the largest airport and major transit hub in the country. The total number of coronavirus disease 2019 (COVID-19)-related deaths during our sampling period in Attica equalled 40% of the total deaths across the country, suggesting that SARS-CoV-2 cases were distributed proportionally across Attica and the rest of Greece. To investigate the patterns of SARS-CoV-2 infection in the areas of northeastern Greece and Thessaly, where virus surges were reported in March and May, respectively, we analyzed 17 samples drawn from Alexandroupoli, Kavala, Komotini, and Xanthi in northeastern Greece and 13 samples from the Nea Smirni area in Larissa, Thessaly (see Fig. S1 in the supplemental material). A few samples (*n* = 4) analyzed as part of routine diagnostic testing in Attica were also available from three Aegean islands (Fig. S1).

10.1128/mSphere.00180-21.1 FIG S1 The sampling areas of SARS-CoV-2 sequences are indicated by red dots across Greece. Download FIG S1, TIF file, 5.6 MB. Copyright © 2021 Kostaki et al. 2021 Kostaki et al. https://creativecommons.org/licenses/by/4.0/ This content is distributed under the terms of the Creative Commons Attribution 4.0 International license.

As mentioned in Materials and Methods, the sampling process covered three time periods. The study samples included 156 of 1,565 diagnosed cases (10%) for the first period, 101 of 1,873 cases (5.4%) for the second period, and 132 of 15,869 cases (0.8%) for the third period. The lower proportion for the third period was due to the number of tests performed increasing gradually with time (i.e., the average number of tests per month was approximately 10× higher in the third period versus the first period), suggesting that the last period was not underrepresented in our sample. To estimate the proportion of samples per the actual number of infections, we used as a proxy the total number of COVID-19-associated deaths estimated for each time period plus 11 days, which is the median time of death since the diagnosis date (https://eody.gov.gr). Specifically, the ratio of samples per deaths were 156 of 90 deaths (1.73) for the first period, 101 of 91 deaths (1.11) for the second period, and 132 of 244 deaths (0.54) for the third period, suggesting that sampling size compared to the total number of deaths was 3 and 2 times higher in the first and second period than the third period, respectively. These differences were smaller than the differences in proportions estimated using the number of diagnosed cases.

The results of the classification of viral sequences into lineages, as estimated using the pangolin program, are presented in Table 1. The most frequent lineages were B.1.1 (European lineage, 40.6%), B.1.1.152 (Russian lineage, 19.5%), B1.1.38 (the United Kingdom lineage, 11.8%), B.1 (a European lineage that corresponds to the spring outbreak in Italy, 5.7%), B (basal lineage from China with many global exports, 4.4%), and B.40 (lineage dominant in the United Kingdom and Australia, 5.1%), while the A lineages originally detected at the early stages of the pandemic in Asia were present at low frequencies in Greece (A2, 0.5%; A5, 0.8%).

**TABLE 1:** Lineages of the study sequences per time period

Lineage	No. of sequences (%) in the following time period:			
	First	Second	Third	All (total)
A.2	2 (1.28)			2 (0.51)
A.5	3 (1.92)			3 (0.77)
B	14 (8.97)	3 (2.97)		17 (4.37)
B.1	13 (8.33)	2 (1.98)	7 (5.3)	22 (5.66)
B.1.1	81 (51.92)	50 (49.5)	27 (20.45)	158 (40.62)
B.1.1.1	1 (0.64)		1 (0.76)	2 (0.51)
B.1.1.38	5 (3.21)	13 (12.87)	28 (21.21)	46 (11.83)
B.1.1.70			1 (0.76)	1 (0.26)
B.1.1.100			1 (0.76)	1 (0.26)
B.1.1.102			1 (0.76)	1 (0.26)
B.1.1.145	1 (0.64)			1 (0.26)
B.1.1.152	8 (5.13)	23 (22.77)	45 (34.09)	76 (19.54)
B.1.1.237		1 (0.99)	2 (1.52)	3 (0.77)
B.1.1.291			9 (6.82)	9 (2.31)
B.1.1.315			1 (0.76)	1 (0.26)
B.1.5	4 (2.56)	1 (0.99)	2 (1.52)	7 (1.80)
B.1.22		1 (0.99)		1 (0.26)
B.1.36			3 (2.27)	3 (0.77)
B.1.36.6			1 (0.76)	1 (0.26)
B.1.98	2 (1.28)			2 (0.51)
B.1.160			1 (0.76)	1 (0.26)
B.1.177			1 (0.76)	1 (0.26)
B.1.255	4 (2.56)			4 (1.03)
B.1.319			1 (0.76)	1 (0.26)
B.3	2 (1.28)			2 (0.51)
B.4	1 (0.64)			1 (0.26)
B.28	1 (0.64)			1 (0.26)
B.39	1 (0.64)			1 (0.26)
B.40	13 (8.33)	7 (6.93)		20 (5.14)

Total	156 (100)	101 (100)	132 (100)	389 (100)

**FIG 1:**
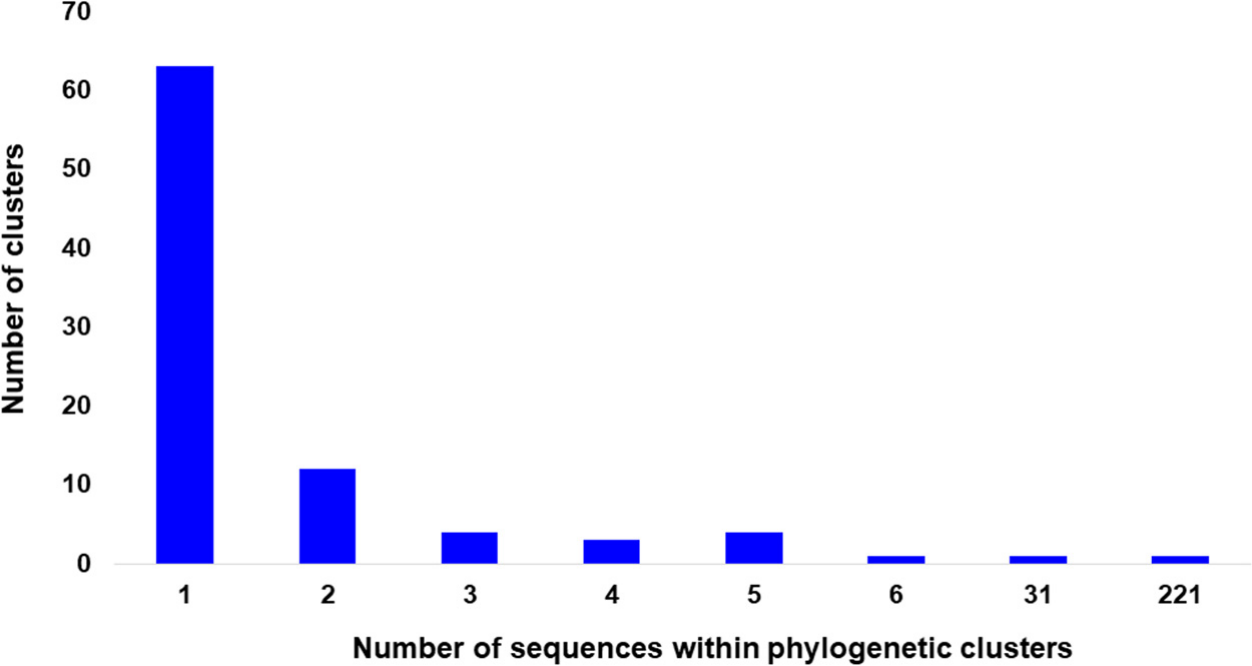
Distribution of the number of sequences per phylogenetic cluster in Greece. The horizontal axis indicates the number of sequences within clusters, and the vertical axis indicates the number of the corresponding clusters.

**FIG 2:**
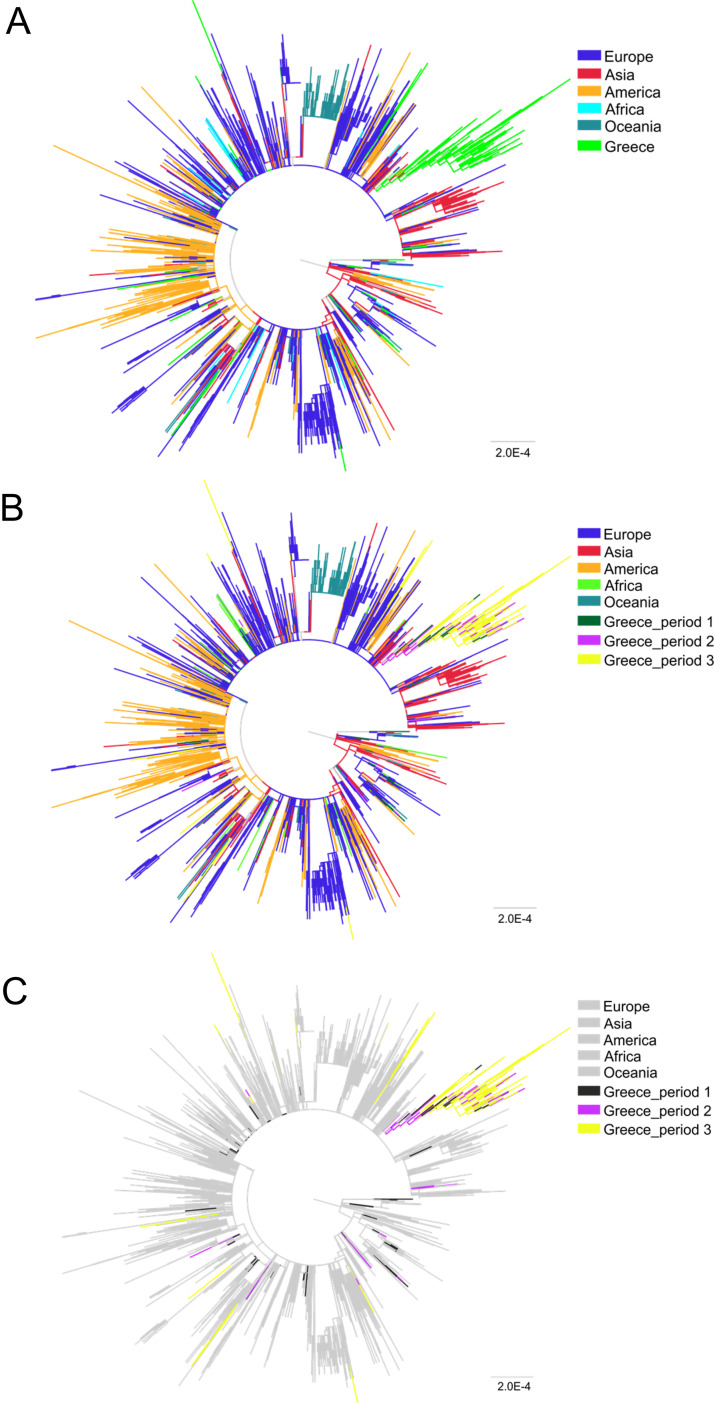
Unrooted phylogenetic tree estimated by FastTree (version 2) of SARS-CoV-2 sequences from Greece (*n* = 389) and a global reference data set *n* = 4,647). (A) All sequences from Greece are colored in light green. (B) Sequences from Greece are marked in dark green (sampling period 1, 29/2/2020 to 31/03/2020 [no travel restrictions]), purple (sampling period 2, 1/4/2020 to 30/6/2020 [travel restrictions]), and yellow (sampling period 3, 1/7/2020 to 29/9/2020 [lifting of travel restrictions]). (C) Sequences from Greece sampled from different time periods are shown in different colors, and all reference sequences are shown in gray.

To investigate the patterns of SARS-CoV-2 pandemic dispersal in Greece, we performed phylogenetic analyses on five different data sets, including as reference a random collection of globally sampled sequences and the best hits identified by BLAST. The selection of sequences was performed over all SARS-CoV-2 lineages to avoid any biases stemming from lineage classification. Phylogenetic analyses on the different alignments revealed similar results, with at least 89 genetically distinct viruses identified as independent introductions in Greece. This number corresponds to the number of sequences (*n* = 63) not falling within phylogenetic clusters with other sequences from Greece, here named singletons, as previously reported (10), plus the number of different clusters (*n* = 26) comprising sequences from Greece. Given that our sample pool corresponds to 10% of the diagnosed cases and, also, that the actual number of SARS-CoV-2 infections was likely severely underdiagnosed, the number of different lineages introduced to Greece should be higher than our estimation. The characteristics of SARS-CoV-2 phylogenetic clusters are depicted in Fig. 1, where, in addition to the 63 sequences that were not associated with onward transmission in Greece, we found several small clusters consisting of 2 to 6 sequences and two larger ones of 31 and 221 sequences (Fig. 2A to C). The second largest cluster included 31 identical sequences sampled at the early stage of the pandemic from 10 to 31 March 2020 in Greece. The largest cluster consisted of 37 (16.7%; first period), 72 (32.6%; second period), and 112 (50.7%; third period) sequences collected during the respective sampling periods (Fig. 2C). Notably, samples from the second and third periods dominated in the largest local cluster. Furthermore, this cluster was underpinned by high levels of phylogenetic support (Shimodaira-Hasegawa [SH] support > 0.9) and was similarly detected in the phylogenetic tree performed using the random sampling of 15,000 GISAID sequences (SH support > 0.85). Specifically, in the latter tree, the composition of clusters was almost identical, with 31 and 217 sequences falling within the two largest clusters.

**FIG 3:**
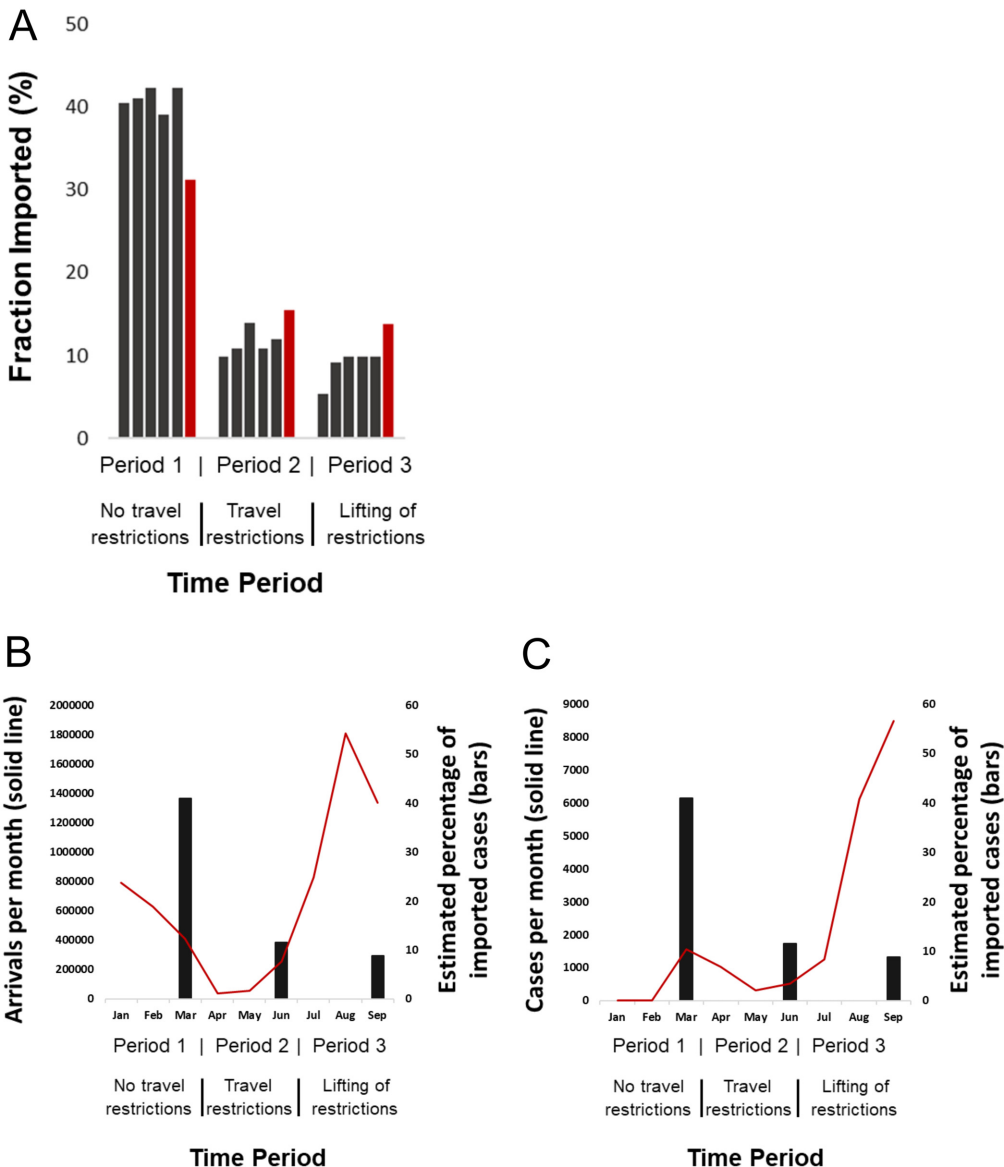
Proportion of virus importation estimated by phylogeographic analysis over the three sampling periods (sampling period 1, 29/2/2020 to 31/3/2020 [no travel restrictions]; sampling period 2, 1/4/2020 to 30/06/2020 [travel restrictions]; sampling period 3, 1/7/2020 to 29/9/2020 [lifting of restrictions]). (A) Proportions of virus importation inferred by phylogeographic analysis using five different data sets (black) and surveillance data (red). Black bars indicate the proportion of virus importation inferred by phylogeographic analysis (mean value estimated from the five different data sets) in combination with the number of international arrivals per month (red line) (B) and the number of SARS-CoV-2 cases per month in Greece (red line) (C).

The putative numbers and sources of virus importation during the three time periods were inferred by means of phylogeographic analyses. The patterns of SARS-CoV-2 importation differed greatly between the three time periods: the proportion of imported infections peaked during the first period (mean value over the five data sets, 41%), while it remained low in the second (mean value over the five data sets, 11.5%) and third (mean value over the five data sets, 8.8%) periods (Fig. 3A). The numbers of imported infections were similar across the different data sets and matched the proportion of imported cases reported from SARS-CoV-2 surveillance (Fig. 3A). The corresponding figures were 31.2%, 15.5%, and 13.8% for the three periods, respectively (Fig. 3A). Implementation of travel restrictions and quarantine measures were applied in the middle of March, causing a decline in international arrivals, and were maintained until June (Fig. 3B). Notably, the proportion of imported infections remained low after the lifting of restrictions on international travel implemented on 1 July in Greece (Fig. 3B), and although a virus surge was detected in August, it was not associated with an increased proportion of imported infections (Fig. 3C).

To investigate the significance of the pattern of virus importation, we compared the previous estimates with the expected proportions of imported infections under a scenario of panmixis. The results of our analysis suggested that the estimated proportions of imported infections were much lower than those expected by chance (Fig. S2), even during the first period (*P* < 0.001); however, the differences were more pronounced in the second and third periods (*P* < 0.001) (Fig. S2). These findings suggest that local transmission eventually dominated during the SARS-CoV-2 pandemic but was less pronounced at the early stages when travel restrictions had not yet been implemented.

10.1128/mSphere.00180-21.2 FIG S2 Proportion of virus importation estimated by phylogeographic analysis over the three sampling periods (sampling period 1, 29/2/2020 to 31/3/2020 [no travel restrictions]; sampling period 2, 1/4/2020 to 30/6/2020 [travel restrictions]; sampling period 3, 1/7/2020 to 29/9/2020 [lifting of travel restrictions]) (red dots) and after simulations of a scenario of panmixis (black dots). Download FIG S2, TIF file, 1.1 MB. Copyright © 2021 Kostaki et al. 2021 Kostaki et al. https://creativecommons.org/licenses/by/4.0/ This content is distributed under the terms of the Creative Commons Attribution 4.0 International license.

The putative geographic origin of the imported infections showed that the majority originated from Europe and specifically from the United Kingdom (23 out of 43 imported cases; 53.5%) in the first period (Table 2). However, a considerable number of transmissions originated from non-European countries (17 out of 43 imported cases; 39.5%) (Table 2). Subsequent analysis revealed that these cases were imported from America and Asia. Specifically, 71.4% of the imported infections outside Europe were from the United States, 14.3% were from Japan, and the remaining 14.3% were from Malaysia. During the second period, most of the imported infections were inferred to have originated from non-European countries (8 out of 9; 88.9%), specifically from Japan, and the rest from the United Kingdom (1 out of 9; 11.1%) (Table 2). During the third period, half of the imported cases were traced to countries outside Europe (66.7% from the United States and 33.3% from Saudi Arabia), and the remaining 33.3% and 16.7% were from the United Kingdom and Denmark, respectively (Table 2). According to the surveillance data, the highest number of imported SARS-CoV-2 cases were from the United Kingdom, at 36.1% and 16.8% for the first and second periods, respectively, proportions that were similar to those estimated by phylogeographic analysis. Data about individuals’ travel history were collected from interviews during sample collection. No information about the origin of potential imported cases was available for the third period.

**TABLE 2:** Estimated number of imported cases (migration events)

Country	No. of imported cases in the following time period:		
	Period 1 (Feb. 29 to Mar. 31) (*n* = 156 sequences)	Period 2 (Apr. 1 to June 30) (*n* = 101 sequences)	Period 3 (July 1 to Sept. 29) (*n* = 132 sequences)
Non-European countries	17	8	3
United Kingdom	23	1	2
Denmark	0	0	1
Germany	3	0	0

**FIG 4:**
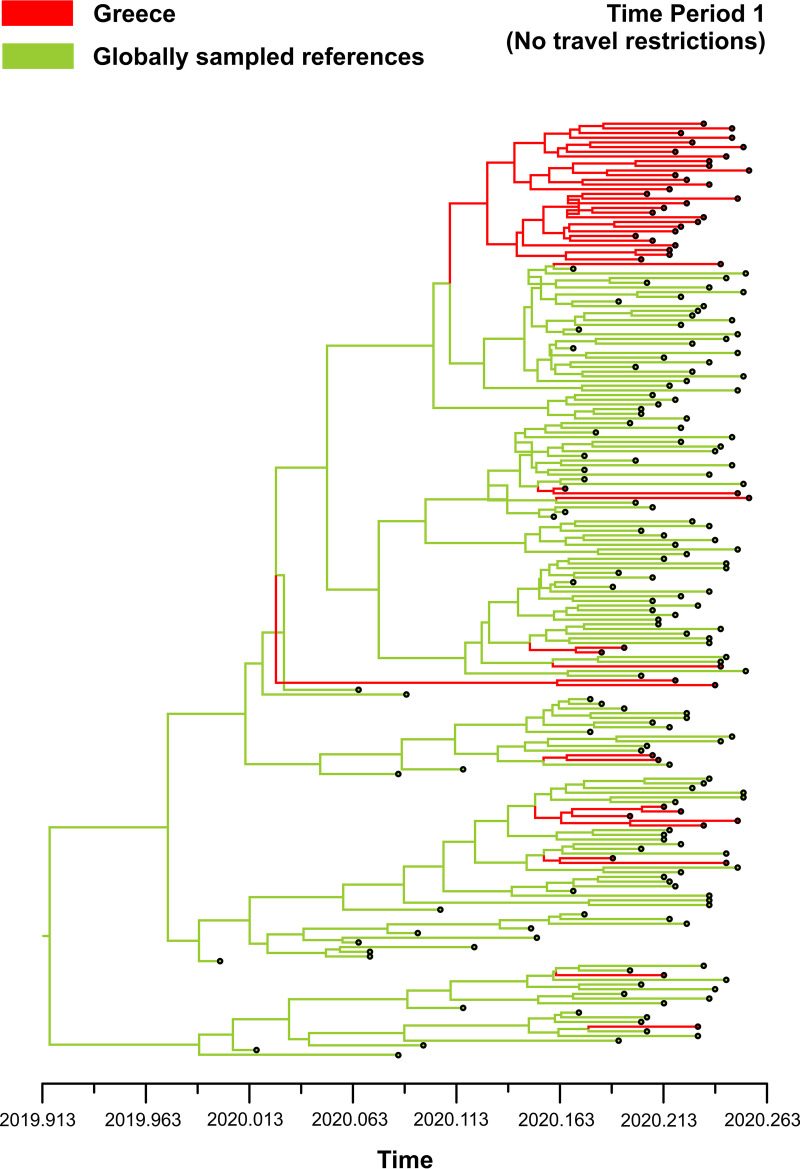
Dated phylogenetic tree of the first sampling phase until the end of March 2020. The time of the most recent common ancestor (tMRCA) of the largest monophyletic cluster including SARS-CoV-2 sequences from Greece is indicated at the node of the corresponding cluster. The monophyletic cluster is shown in red, and reference sequences are shown in green.

The patterns of imported infections in the three time periods were verified using a Bayesian phylogeographic approach on three different data sets, including 50 sequences from Greece and 150 globally sampled reference sequences. The median number of imported infections was 13.9 (95 highest posterior density [HPD], 11.0 to 17.3) corresponding to 27.8%, 6.2 (95 HPD, 5.0 to 7.1) corresponding to 12.4%, and 5.2 (95 HPD, 5.0 to 6.0) corresponding to 10.4% for the three time periods, respectively. These figures were similar to our previous phylogeography inferences using the criterion of parsimony and confirmed that viral dispersal pattern differed between the three time periods. Based on the dated tree of the first sampling period corresponding until the end of March 2020, the time of origin of the most recent common ancestor (tMRCA) of the largest monophyletic cluster of SARS-CoV-2 sequences from Greece was estimated to be 15 February 2020 (95% HPD, 2 February 2020 to 26 February 2020] (Fig. 4). This date corresponds to the putative origin of SARS-CoV-2 infections in Greece.

## DISCUSSION

In the current study, using SARS-CoV-2 genomes from three distinct time periods, we showed that imported lineages were responsible for 41% of transmissions during the first pandemic wave in Greece. Moreover, we found that levels of virus importation significantly decreased during the period of travel restrictions and quarantine measures and, notably, remained low even after the opening of borders during the 3 months of the peak tourist season. Our results were robust across different reference data sets and correlated strongly with the surveillance data regarding both the proportion of imported infections and the putative origin of SARS-CoV-2 lineages. The transmission patterns were further confirmed using a Bayesian phylogeographic approach and although the analysis was performed after downsampling, the pattern of imported infections was similar to the statistical phylogeography estimations, with the number of imported cases being lower in the period of travel ban and quarantine and after the lifting of travel restrictions. Our findings suggest that imported infections dominated at the early stage of the pandemic before the implementation of travel bans.

More importantly, we found that virus importation remained low and did not substantially contribute to SARS-CoV-2 onward transmission even after the lifting of travel restrictions. Since 1 July 2020, all incoming travelers, including Greek citizens, need to have completed a passenger locator form (PLF) 48 h before entering Greece. Health screening procedures have been put in place at airports and other ports of entry, where targeted testing has been performed guided by an artificial intelligence algorithm termed EVA. The algorithm combines information from previous tests performed at entry points in the country, as well as data obtained from the PLFs, creating an importation risk profile for each visitor according to the country of origin. Health authorities can utilize this profile to determine border molecular testing prioritization, thus enhancing public health protection. Risk assessment for all countries was continuously performed daily, and measures were modified accordingly, such as when entry restrictions were tightened for some countries when an increase in the number of SARS-CoV-2-positive cases was observed (a negative SARS-CoV-2 PCR test being required for entry). Additional public health measures were implemented (social distancing, local lockdowns, compulsory use of face masks in public spaces, etc.) locally or nationwide as necessary by local and national authorities.

Our study suggests that the impact of travelers to SARS-CoV-2 local transmission in Greece was low during the summer. To our knowledge, this is one of few molecular epidemiology studies showing that the lifting of travel restrictions after the first pandemic wave was not associated with onward transmission driven by imported SARS-CoV-2 cases. This was most likely due to virus screening at entry points and public health measures implemented during the summertime and afterwards, which helped to control virus spread in the community. Notably, except for a few islands (i.e., Paros, Mykonos), no virus surges were detected during the summer period in Greece, and the effective reproductive number R remained around 1.1 to 1.2 during this period (National Public Health Organization, unpublished data).

Early importation events observed during the first period resulted in large clusters only in one case; local clusters, potentially seeded after the lifting of travel restrictions, may have remained undetected in our study. However, if this hypothesis were true, we would expect to have observed a high number of singleton (imported) infections, which was not the case. This suggests that the former hypothesis does not provide the most plausible explanation for the SARS-CoV-2 dispersal pattern during the third described period in Greece. Moreover, although our samples were not collected at tourist destinations, they were drawn from Attica, where almost 40% of the total Greek population resides, and which fuels tourism in these destinations during the summertime. Therefore, if new strains were associated with high levels of local transmission, we should have been able to detect them through our sampling.

Our findings are similar to those of previous studies in Europe and the Americas showing that the levels of imported infections declined after the implementation of travel restrictions during the first pandemic wave (10). The scale of virus importation in Greece was in accordance with that in Boston, Massachusetts, prior to 28 March 2020 (approximately 35%) and thereafter, when travel restrictions were implemented (median, 9.3%) (7). We also showed that the majority of strains during the first pandemic wave were imported from Europe, and specifically from the United Kingdom, but a significant proportion of virus importation originated in non-European countries. This pattern matched the origin of cases associated with travel during the first phase, suggesting that, although phylogeographic accuracy can be compromised due to potentially nonrepresentative sequencing, in our analyses, the putative origin of imported cases estimated by phylogeography matched that estimated by surveillance data.

Bayesian phylogeographic analysis revealed that the tMRCA of the largest SARS-CoV-2 was 15 February 2020, considered the putative origin of SARS-CoV-2 infections in Greece. This date precedes the first case diagnosed in 26 February 2020 in Greece, suggesting that virus was circulating for at least 10 days before it became diagnosed. Although the hypothesis for earlier introduction cannot be rejected, if it happened, it was most likely at low levels and was probably not associated with onward transmission in the population.

Our study has several limitations. Our sampling was not representative and was not performed across Greece. On the other hand, as discussed above, our analysis was based on 389 full-length genomes collected at different time points from Attica, Greece, suggesting that our results reflect a large proportion of the population. Moreover, our study data included a dense sample of diagnosed cases during the first and second phases; sampling proportion was lower in the third period, but this was due to the enhanced testing performed over time. In addition, if viral transmission pattern had been different during the second and third phases (i.e., the number of imported cases had remained as high as in the first phase), we would have been able to identify these changes even with lower sampling proportions. Notably, the proportion of imported cases remained robust after a downsampling and using a Bayesian approach, thus suggesting that viral transmission patterns can be inferred even with lower sampling. Regarding the putative limitation of nonsampling from tourist destinations during the third phase, if SARS-CoV-2 was continuously transmitted from viral lineages imported during the summertime in Greece, we would be able to detect them in Attica residents, a large proportion of whom visit different places in Greece during the summertime. Our findings suggest that imported cases did not contribute substantially to SARS-CoV-2 local spread between July and September 2020. Importantly, our results on the effects of virus importation correlated with those estimated from surveillance data, thus enhancing the robustness of our findings. We should note that our findings are relevant to the summer period in the Mediterranean region and may not be generalizable for areas with different climatic conditions.

In conclusion, our molecular epidemiology study showed that the estimated proportion of imported cases during the first pandemic wave in Greece was 41% and that virus screening and public health measures after the lifting of travel restrictions prevented SARS-CoV-2 onward transmission from imported cases during summer 2020. These findings provide important insights on the efficacy of targeted public health measures and have important implications regarding the safety of international travel during a pandemic.

## MATERIALS AND METHODS

### Analyzed SARS-CoV-2 samples.

The SARS-CoV-2 samples analyzed in the context of the current study were collected from 29 February to 19 September 2020 in the Attica, Larisa, and Thrace regions of Greece, from two SARS-CoV-2 reference centers. Specifically, all SARS-CoV-2-positive samples available with reverse transcription-PCR (RT-PCR) threshold detection cycles (*C_T_*) ≤ 30 until the end of August 2020 from Attikon University Hospital (first reference center) were included in our analysis. Similarly, samples fulfilling the previous criterion available in September 2020 at the Department of Hygiene, Epidemiology and Medical Statistics of the School of Medicine at National and Kapodistrian University of Athens (second reference center) were included in our analysis. SARS-CoV-2 samples obtained at border control areas from travelers arriving in Greece were excluded from the analysis. Specimen types included rhino-oropharyngeal swabs. Sample inactivation/RNA extraction was performed according to different protocols available across the different laboratories from which the RNA samples were made available.

The study was approved by the Ethics and Bioethics Committee of the Medical School of the National and Kapodistrian University of Athens (protocol 300/25-05-2020).

The SARS-CoV-2 samples selected were part of the routine diagnostic procedures performed in Greece at the two reference centers in Attica or elsewhere in Greece. SARS-CoV-2 testing in Greece is prioritized for symptomatic individuals, high-risk contacts of SARS-CoV-2-positive cases, or populations at high risk for SARS-CoV-2 infection (i.e., health care workers, elderly and/or disabled nursing home residents). Samples collected in September included those performed by the National Public Health Organization (https://eody.gov.gr/en/) as part of volunteer testing of the population in Attica, Greece.

Our sampling comprised three time periods: between 29 February and 31 March 31 2020, between 1 April and 30 June 2020, and between 1 July and 29 September 2020. The three time periods were defined according to the status of travel restrictions implemented in Greece during the SARS-CoV-2 pandemic. Specifically, a general travel ban and quarantine measures (i.e., 14-day quarantine) for all travellers upon arrival from abroad were implemented on March 14 and 16, respectively, and, given the length of the SARS-CoV-2 incubation period, the first phase was extended until the end of March 2020. Similarly, restrictions on all nonessential movement throughout the country were implemented on 23 March 2020. The second period corresponded to when the international travel restrictions were in place, including a quarantine period for arriving travellers, and the third corresponded to the period after the lifting of travel restrictions, based on a thorough risk assessment for all international travellers.

### Next-generation sequencing (NGS).

RNA samples were processed using the CleanPlex SARS-CoV-2 panel (Paragon Genomics) according to the manufacturer’s instructions. Samples were quantitated (Qubit RNA HS [high-sensitivity] assay kit; Thermofisher), and 50 to 100 ng of total RNA was used for library preparation, with a final PCR amplification of 24 to 26 cycles. The resulting libraries were analyzed on a Bioanalyzer system (high-sensitivity DNA kit; Agilent), quantified (Qubit double-stranded DNA [dsDNA] HS assay kit; Thermofisher), and multiplexed; they were sequenced on a NextSeq 550 System (Illumina), using the Mid Output kit v2.5 (300 cycles), in paired-end mode.

The quality of FASTQ files was assessed using FastQC (version 0.11.9) (18). Prior to more exhaustive quality controls, the workflow recommended by Paragon Genomics (for full details, see https://github.com/moulos-lab/greek-covid19-assembly) was applied for adapter and primer sequence trimming. Further potential adapter leftovers and poor-quality bases were trimmed with TrimGalore (version 0.6.6) (19), which also deploys Cutadapt (version 2.8) (20), keeping reads with length of at least 50 bp. Subsequently, the remaining paired short reads were normalized to 100× uniform coverage using BBnorm from the BBmap suite (21) and then subjected to guided *de novo* genome assembly using SPAdes (version 3.14.1) (22) with the *--careful* option. The guided SARS-CoV-2 genome assembly is achieved by using a reference genome with the –trusted-contigs option of SPAdes. The reference SARS-CoV-2 genome for the guided assembly was retrieved from University of California at Santa Cruz (23). The quality of the assemblies was assessed using QUAST (version 5.0.2) (24). By using the guided approach, the vast majority of the assemblies was complete. For the few assemblies that were not complete, the MEDUSA scaffolder was deployed in order to complete the assemblies (25). The short reads were also mapped to the SARS-CoV-2 genome retrieved from UCSC using BWA (version 0.7.17) (26) in order to further assess the quality of virus sequencing and visually inspect coverage and potential virus mutations.

The complete *de novo* genome assembly and assessment procedure can be found online (https://github.com/moulos-lab/greek-covid19-assembly). The bioinformatics analysis was performed using the computational infrastructure of the Center of New Biotechnologies & Precision Medicine (pMedGR), School of Medicine, National and Kapodistrian University of Athens, Greece (https://www.precisionmedicine.gr/).

### Sequence alignment and phylogenetic analysis.

The dispersal patterns of SARS-CoV-2 in Greece were investigated by means of phylogenetic analysis. Classification of SARS-CoV-2 sequences in different lineages was implemented in the pangolin webtool (https://cov-lineages.org/pangolin.html). Our data consisted of the following: (i) five data sets of 3,000 randomly selected sequences until 30 September 2020 sampled from the GISAID database plus the best hits identified by a BLAST search using as queries all sequences of our study population against the GISAID database sampled at the same time period, and (ii) a data set of 15,000 randomly selected sequences sampled until 30 September 2020 plus the best hits identified by a BLAST search. We collected 173,991 sequences of high quality and of length >29.000 nucleotides (nt) from GISAID until 30 September 2020 and created a BLAST Database. We queried the 389 sequences and set a threshold to report the first 50 best hits (ranked by E value and bitscore). BLAST reported pairwise 20,517 hits matching at different regions. These hits correspond to 2,059 unique sequences. The BLAST search was performed using only the coding region of SARS-CoV-2 (29,410 nt). The total sizes of unique sequence data sets after the inclusion of the best hits, the random sets of 3,000 sequences, and our study population, were 5,039 (data set 1), 5,036 (data set 2), 5,038 (data set 3), 5,039 (data set 4), and 5,036 (data set 5). The size of the data set including the 15,000 randomly selected sequences was 16,919 unique sequences. Analysis was performed without taking into account the classification of SARS-COV-2 into lineages.

Multiple sequence alignments were performed using the multithreaded version of the MAFFT program (27). This was run using XSEDE, available from the cyberinfrastructure for phylogenetic research (The CIPRES Science Gateway, version 3.3; https://www.phylo.org/), and the infrastructure at pMedGR.

Phylogenetic analyses were carried out by the maximum likelihood (ML) method using the IQ-Tree (version 2.1.1) (28) and the FastTree (version 2) (29) programs. Due to constraints in computation time, phylogenetic trees for sequence alignments larger than 3,000 sequences were imputed using FastTree. Phylogenetic analysis was performed using the best-fit nucleotide substitution with ModelFinder and the Akaike information criterion (AIC) as implemented in IQ-Tree (28). The nucleotide substitution model selected more often was GTR+I+G4. For FastTree, GTR+G4 was selected as the nucleotide substitution model. All runs were performed at the CIPRES Science Gateway (version 3.3) and the infrastructure at pMedGR.

The resulting phylogenies were visualized and annotated by the FigTree (version 1.4) and the Dendroscope (version 3.7.2) programs.

### Estimation of the number of imported SARS-CoV-2 infections.

Τo infer the dispersal patterns of SARS-CoV-2 (i.e., the number of imported infections versus the within country infection events during different time periods), we performed phylogeographic analysis on all five data sets of 5,036 to 5,039 sequences each. Phylogeographic analysis was performed on the ML tree reconstructed by phylogenetic analysis conducted on the FastTree program in the previous analysis step. Specifically, we estimated the number of SARS-CoV-2 infections (viral migration events) between different geographic areas/countries around the world and Greece (imported infections) during three time periods: between (i) 29 February and 31 March 2020, (ii) 1 April and 30 June 2020, and (iii) 1 July 1 and 29 September 2020. Additionally, we estimated infections occurring locally (local infections) between individuals for whom viral samples were taken at these three time periods. The viral migration events were quantified between the different geographic areas/countries by character reconstruction using the criterion of parsimony as implemented in PAUP*4.0 (30).

We assessed whether the inferred migration events (imported or local infections) were different from those expected by chance (panmixis). Hypothesis testing was performed by character reconstruction using the criterion of parsimony on the Mesquite (version 3.61) program (31). Further details on the methodology of viral migration event estimation have been described in detail elsewhere (32–35).

### Bayesian phylogeographic analysis.

We further conducted phylogeographic analysis by using the Bayesian approach to verify our findings. Analysis was performed separately on three data sets representing the three periods of sampling (up to March, April to June, and July to September). Each data set consisted of 200 randomly selected sequences (50 sequences from Greece and 150 globally sampled reference sequences). Analysis was performed using the general time reversible (GTR) nucleotide substitution model with the G heterogeneity model, an uncorrelated log normal relaxed clock of molecular clock model with TipDates, a Bayesian skyline nonparametric plot demographic model with 10 groups, and a discrete phylogeographic approach with a nonreversible continuous-time Markov chain (CTMC) model, a Bayesian stochastic search variable selection (BSSVS), and a Markov jump count parameter (36, 37), as implemented in BEAST v1.8.0 (38). Noninformative priors were used for the Markov chain Monte Carlo (MCMC) runs. MCMC analysis was run for each data set for 100 × 10^6^ to 200 × 10^6^ generations, sampled every 10.000 to 20.000 steps (burn-in, 10%). The MCMC convergence and the effective sample sizes (ESS) were checked using the program Tracer v1.7.1. The maximum clade credibility (MCC) tree was selected from the posterior tree distribution by the TreeAnnotator v1.8 program (38) and visualized by the FigTree v1.3.1 program.
