# Supplementary figures and images for: Molecular Epidemiology of SARS-CoV-2 in Greece Reveals Low Rates of Onward Virus Transmission after Lifting of Travel Restrictions Based on Risk Assessment during Summer 2020
Source: mSphere. 2021 Jun 30;6(3):e00180-21. doi: 10.1128/mSphere.00180-21 (PMC8265632; doi:10.1128/mSphere.00180-21)

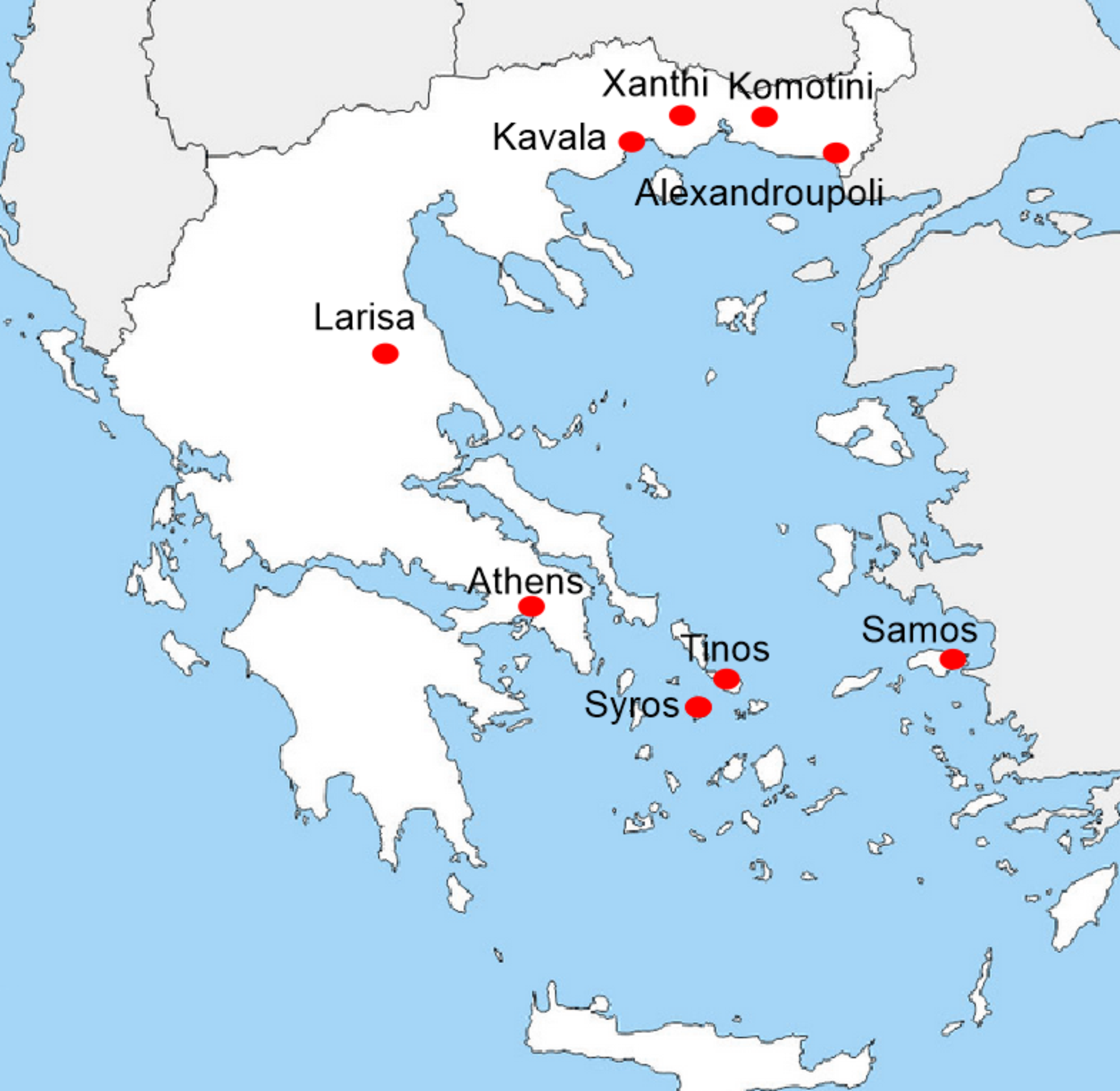

Supplement: FIG S1 [file msphere.00180-21-sf001.tif]

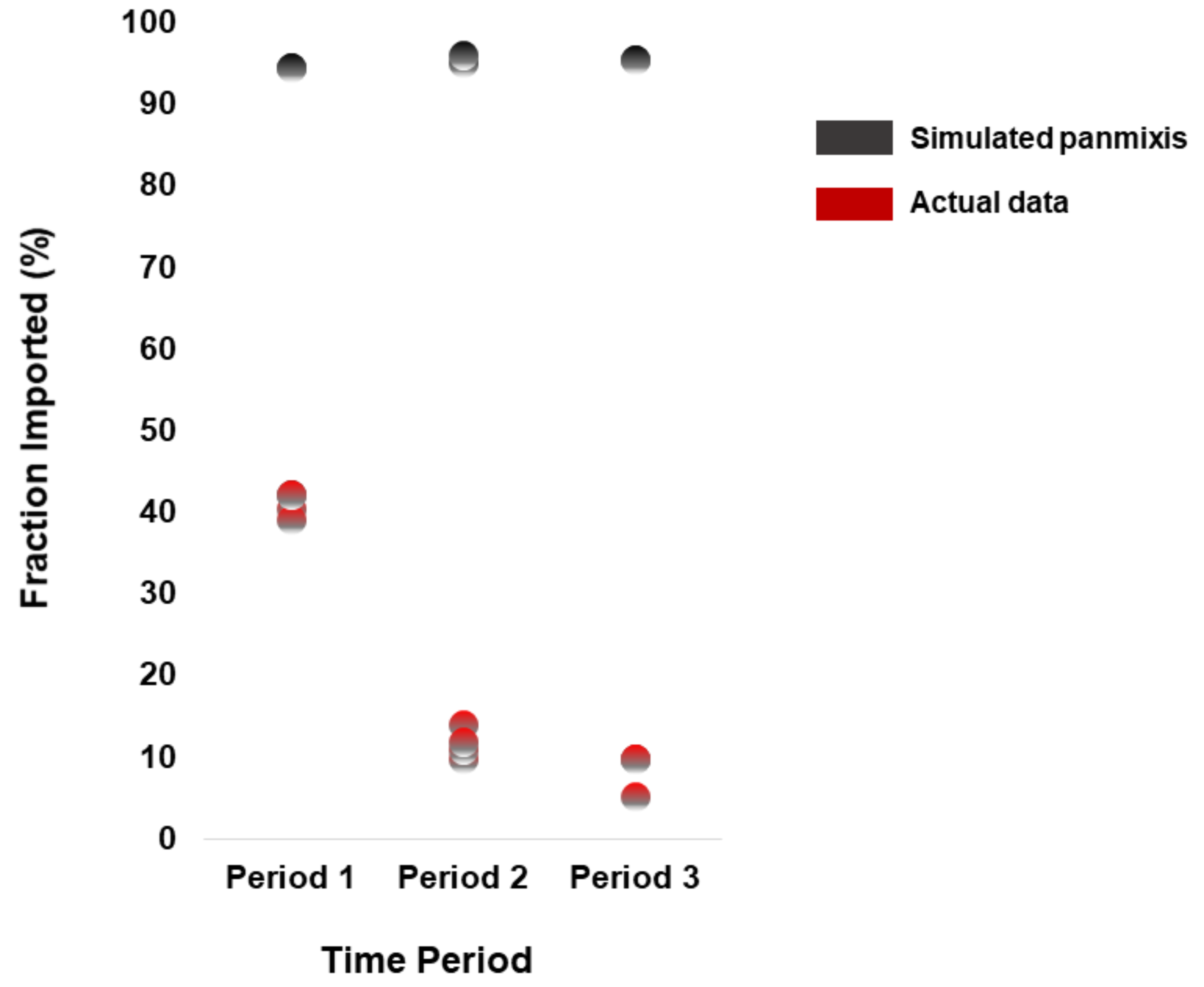

Supplement: FIG S2 [file msphere.00180-21-sf002.tif]
